# Evolutionary conservation of receptor compensation for stem cell homeostasis in Solanaceae plants

**DOI:** 10.1093/hr/uhae126

**Published:** 2024-05-03

**Authors:** Myeong-Gyun Seo, Yoonseo Lim, Anat Hendelman, Gina Robitaille, Hong Kwan Beak, Woo-Jong Hong, Soon Ju Park, Zachary B Lippman, Young-Joon Park, Choon-Tak Kwon

**Affiliations:** Graduate School of Green-Bio Science, Kyung Hee University, Yongin 17104, Republic of Korea; Graduate School of Green-Bio Science, Kyung Hee University, Yongin 17104, Republic of Korea; Cold Spring Harbor Laboratory, Cold Spring Harbor, New York 11724, USA; Howard Hughes Medical Institute, Cold Spring Harbor Laboratory, Cold Spring Harbor, New York 11724, USA; Cold Spring Harbor Laboratory, Cold Spring Harbor, New York 11724, USA; Howard Hughes Medical Institute, Cold Spring Harbor Laboratory, Cold Spring Harbor, New York 11724, USA; Division of Biological Sciences and Research Institute for Basic Science, Wonkwang University, Iksan, Jeonbuk 54538, Republic of Korea; Department of Smart Farm Science, Kyung Hee University, Yongin 17104, Republic of Korea; Division of Applied Life Science, Plant Molecular Biology and Biotechnology Research Center, Gyeongsang National University, Jinju 52828, Republic of Korea; Cold Spring Harbor Laboratory, Cold Spring Harbor, New York 11724, USA; Howard Hughes Medical Institute, Cold Spring Harbor Laboratory, Cold Spring Harbor, New York 11724, USA; Department of Smart Farm Science, Kyung Hee University, Yongin 17104, Republic of Korea; Graduate School of Green-Bio Science, Kyung Hee University, Yongin 17104, Republic of Korea; Department of Smart Farm Science, Kyung Hee University, Yongin 17104, Republic of Korea

## Abstract

Stem cell homeostasis is pivotal for continuous and programmed formation of organs in plants. The precise control of meristem proliferation is mediated by the evolutionarily conserved signaling that encompasses complex interactions among multiple peptide ligands and their receptor-like kinases. Here, we identified compensation mechanisms involving the CLAVATA1 (CLV1) receptor and its paralogs, BARELY ANY MERISTEMs (BAMs), for stem cell proliferation in two Solanaceae species, tomato and groundcherry. Genetic analyses of higher-order mutants deficient in multiple receptor genes, generated via CRISPR-Cas9 genome editing, reveal that tomato *SlBAM1* and *SlBAM2* compensate for *slclv1* mutations. Unlike the compensatory responses between orthologous receptors observed in *Arabidopsis*, tomato *slclv1* mutations do not trigger transcriptional upregulation of four *SlBAM* genes. The compensation mechanisms within receptors are also conserved in groundcherry, and critical amino acid residues of the receptors associated with the physical interaction with peptide ligands are highly conserved in Solanaceae plants. Our findings demonstrate that the evolutionary conservation of both compensation mechanisms and critical coding sequences between receptor-like kinases provides a strong buffering capacity during stem cell homeostasis in tomato and groundcherry.

## Introduction

The plant kingdoms boast abundant duplicate genes resulting from multiple rounds of whole-genome duplication or polyploidization [1, 2]. The significantly diverse genome sizes in plant genomes suggest that many paralogs have taken dynamic evolutionary paths postduplication [3, 4]. While some paralogs have maintained redundant functions, they have also shielded themselves from deleterious mutations through selective pressures, thereby enhancing genetic robustness [5, 6]. The functional overlaps among duplicates further enable genetic compensation, acting as a buffer against null mutations [7–9]. Various duplication events in plant species have led to gene families acquiring more than one member, demonstrating the ability of multiple gene copies to neutralize both genetic and environmental perturbations [2, 8]. One notable example involves receptor-like kinases and peptide ligands, critical for plant development, which are classified into diverse families [10, 11]. These families have dynamically diverged from mosses to angiosperms throughout plant evolution, resulting in a multitude of family members within a species [12–14].

Plants have a distinctive capacity to continually produce new organs during their life cycles. The apical meristems at the expanding shoot and root tips serve as the ongoing origins of organ formation. Within the shoot meristem, plant stem cells represent a group of cells with the remarkable capacity to give rise to entire above-ground organs [15]. The equilibrium between maintaining and differentiating stem cells must be tightly controlled throughout plant growth and development [16]. Thus, understanding the mechanisms governing shoot apical meristem control is crucial for unraveling the complexities of plant development. The signaling pathway involving *WUSCHEL* (*WUS*), *CLAVATA3* (*CLV3*) and *CLAVATA1* (*CLV1*) has evolved as the principal regulatory mechanism that coordinates shoot apical meristem maintenance. [17]. In Brassicaceae species *Arabidopsis thaliana* (*Arabidopsis*), *WUS*, encoding a homeodomain transcription factor, induces *CLV3* expression and stimulates stem cell proliferation [18]. *CLV3* encodes a small signaling peptide recognized by leucine-rich repeat receptor-like kinases (LRR-RLKs), including CLV1 and BARELY ANY MERISTEM (BAM) [19–22]. These LRR-RLKs stabilize with their coreceptor, CLV2 [23]. Activation of CLV3-CLV1 signaling inhibits *WUS* expression, constituting a self-regulatory loop [18]. The negative feedback circuit between *CLV3* and *WUS* is highly conserved in land plants and crucial for the appropriate development of shoot apical meristem [15]. Disrupting CLV3-CLV1 signaling in various species induces stem cell overproliferation, resulting in fasciation phenotypes [17, 24, 25].

CLV3/EMBRYO SURROUNDING REGION (CLE) peptides and their receptors belong to structurally conserved gene families, but their functions are not entirely identical [26], suggesting complex genetic redundancy among these genes. In Solanaceae species *Solanum lycopersicum* (tomato), *slclv3* mutants showed enlarged shoot apical meristems and extra floral organs, and fasciation was dramatically enhanced in *slclv3 slcle9* mutants [27]. Notably, transcription of *SlCLE9* is upregulated in *slclv3* null mutants, indicating a mechanism of ‘active paralogous compensation’ characterized by immediate gene expression alterations following the functional impairment of its paralog *in vivo* [8, 27]. In *Arabidopsis*, multiple *CLE* members can mitigate the *clv3* mutant phenotype without altering their expression levels, indicating a ‘passive paralogous compensation’ mechanism that requires no molecular changes to substitute for the function of the lost paralog [8, 27]. The nonlinear dynamics between gene expression and redundant functional activity mean that removing one paralog could halve protein levels but only slightly affect their collective function, leading to minimal phenotypic alterations [8].

We previously demonstrated that the strength of compensation is determined by variations in both the coding region of dodecapeptides and their expression during conserved active compensation of peptide ligands in tomato, *Physalis grisea* (groundcherry), and *Petunia hybrida* (petunia) [28]. This indicates that buffering systems of meristem homeostasis are diversified, while the core module of CLV-WUS signaling is highly conserved in Solanaceae plants. In addition to compensation mechanisms between peptide ligands during meristem maintenance, there are buffering systems between CLV1 and its closest paralog BAM receptors [21, 29]. The phenotypes of *Arabidopsis clv1* mutants are actively compensated by derepressed *BAM* genes [30]. However, it remains unexplored whether compensation mechanisms between peptide receptors during stem cell proliferation are diversified in Solanaceae plants.

In this study, we generated single and higher-order mutants deficient in *CLV1* and *BAM* homologous genes using CRISPR-Cas9 to examine genetic compensation in tomato and groundcherry. The severity of floral fasciation in both *slclv1* and *pgclv1* mutants was mitigated by BAM receptors, while *slbam1 slbam2* and *pgbam1 pgbam2* double mutants resembled wild-type plants. Notably, none of the *SlBAM* and *PgBAM* genes significantly increased in *slclv1* and *pgclv1* mutants, suggesting passive compensation between peptide receptors during stem cell proliferation in tomato and groundcherry. Additionally, critical amino acid residues of CLV1 and BAM receptors associated with physical interaction with CLV3 and CLE dodecapeptides are nearly conserved in Solanaceae. Our findings show that strong passive compensation between receptor paralogs in tomato and groundcherry enables partial perception of derepressed peptide ligands when a part of receptor signaling is weakened, providing buffering capacity for stem cell homeostasis.

## Results

### Phylogenetic and expression analyses of *SlCLV1* and its paralogs

**Figure 1:**
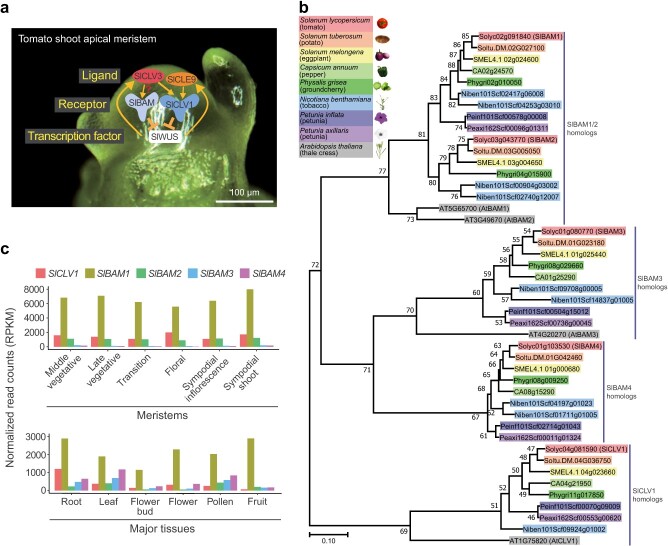
Phylogenetic and expressional analyses of *SlCLV1* and its paralogs. (a) Schematic model of conserved molecular pathway for meristem proliferation in tomato. SlCLV3 and SlCLE9 are small signaling peptides acting as ligands. SlBAM and SlCLV1 are receptors that interact with ligands to repress gene expression of the downstream transcription factor, SlWUS. A question mark indicates that the association between SlCLV3 and SlBAM remains to be determined. (b) Phylogenetic analysis of *SlCLV1* and *SlBAM* homologs in Solanaceae plants and *Arabidopsis*. Bootstrap values from 1000 replicates are presented on each node. **c** Normalized expression for *SlCLV1* and its paralogs, *SlBAM1*, *SlBAM2*, *SlBAM3*, and *SlBAM4*, in meristems and major tissues. RPKM, reads per kilobase of transcript per million mapped reads. At least twice experiments were repeated independently with similar results.

Stem cell homeostasis is tightly controlled by multiple peptides and their receptors in tomato shoot apical meristem [27]. The SlCLV3 dodecapeptide serves as a ligand binding to the receptor-like kinase SlCLV1 and possibly its paralog SlBAMs, which negatively regulate *SlWUS* transcription to promote meristem proliferation (Fig. 1A). SlWUS enhances the transcription of *SlCLV3* and its paralog *SlCLE9* [31], constituting a conserved negative feedback loop during stem cell control (Fig. 1A). In contrast to SlCLV3, perception of the SlCLE9 dodecapeptide is mainly mediated by SlCLV1 (Fig. 1A) [27].

To understand how receptor compensation for meristem homeostasis evolves, we investigated the homologs of major receptor genes, *CLV1* and *BAMs*, in Solanaceae species. Our analysis of the Solanaceae genome revealed that homologs of *CLV1* and *BAM* genes are conserved in tomato, *Solanum tuberosum* (potato), *Solanum melongena* (eggplant), *Capsicum annuum* (pepper), groundcherry, *Nicotiana benthamiana* (tobacco), and petunia (Fig. 1B, Supplementary Table S1). One *SlCLV1* and four *SlBAM* homologs are conserved in tomato, potato, eggplant, and groundcherry, whereas pepper, petunia, and *Arabidopsis* harbors one *CLV1* and three *BAM* genes (Fig. 1B). Interestingly, we discovered that the pepper and petunia genome lacks an ortholog of the tomato *SlBAM2*, and allotetraploid tobacco has one *SlCLV1* ortholog (Fig. 1B).

*SlCLV1* and *SlBAM* genes were expressed throughout the whole plant but with different levels depending on the tissue and developmental stage (Fig. 1C, Supplementary Fig. S1, Supplementary Table S2). The *SlBAM1* gene exhibited the highest levels of expression among *SlCLV1* and four *SlBAM* genes (Fig. 1C). In the shoot meristems, *SlBAM3* and *SlBAM4* showed relatively low gene expression compared with *SlCLV1*, *SlBAM1*, and *SlBAM2* (Fig. 1C). However, the transcription levels of *SlBAM3* and *SlBAM4* were higher than that of *SlBAM2* in other tissues such as root, leaf, flower bud, flower, and pollen (Fig. 1C). The tissue-dependent expression of the four *SlBAM* genes indicates unequal genetic redundancy among *SlBAM* genes.

### Fasciation phenotypes of single and double mutants deficient in *SlCLV1* and *SlBAM* genes

**Figure 2:**
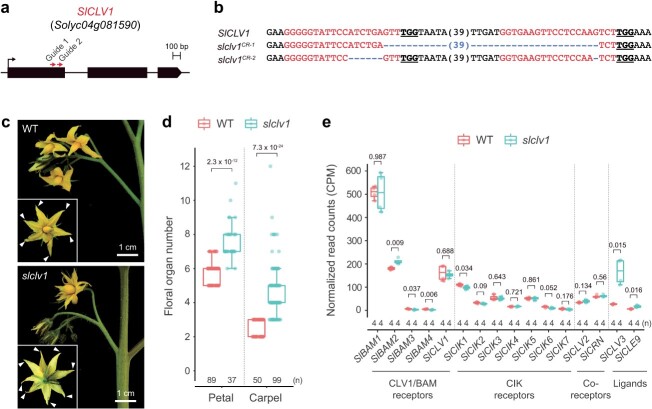
Phenotypic and transcriptome analyses of CRISPR-generated *slclv1* mutants. (a) Gene structures of *SlCLV1*. (b) CRISPR-generated mutations of *SlCLV1*. Protospacer-adjacent motif (PAM) and guide RNA sequences are highlighted. Numbers in parentheses indicate gap lengths. (c) Inflorescence and flower of tomato wild-type (WT) and *slclv1* plants. White arrowheads mark petals. (d) Quantification of floral organ (petal and carpel) numbers of WT and *slclv1* plants. (e) Normalized RNA-seq read counts of *SlBAM1*, *SlBAM2*, *SlBAM3*, *SlBAM4*, *SlCLV1*, *SlCIK1, SlCIK2, SlCIK3, SlCIK4, SlCIK5, SlCIK6, SlCIK7, SlCLV2*, *SlCRN*, *SlCLV3*, and *SlCLE9* from WT and *slclv1* meristems. Each replicate (n) is from 30–40 meristems. CPM, count per million. Box plots, 25th–75th percentile; center line, median; whiskers, full data range. *P* values (two-tailed, two-sample *t*-test) are indicated on the box plots. At least twice experiments were repeated independently with similar results.

For genetic validation of *SlCLV1*-mediated stem cell control, we initially generated *slclv1* null mutants using CRISPR-Cas9 genome editing technology with two guide RNAs (Fig. 2A and B) [27]. The *slclv1* mutants produced more floral organs than wild-type plants (Fig. 2C and D), although their fasciation phenotypes were substantially weaker than those of the *slclv3* single and *slclv3 slcle9* double mutants [27, 28]. These observations suggest that SlCLV1 is not solely responsible for the perception of SlCLV3 and perception of SlCLV3 by other receptor(s) alleviates the severity of *slclv1* mutants. Transcription of *SlCLV3* and *SlCLE9* was significantly induced in *slclv1* mutants, consistent with the conserved negative feedback loop during meristem homeostasis (Fig. 2E). In contrast, none of *SlBAM* genes increased by more than 2-fold in *slclv1* mutants, unlike the approximately 6-fold and 3-fold increases in *SlCLV3* and *SlCLE9*, respectively (Fig. 2E, Supplementary Table S2). In addition, the loss of *SlCLV1* did not significantly influence the expression of tomato *CLAVATA3 INSENSITIVE RECEPTOR KINASEs* (*SlCIKs*), *SlCLV2*, and *SlCORYNE* (*SlCRN*), which encode either receptors or coreceptors for peptide ligands [12, 32, 33]. Therefore, active compensation mechanisms between receptor-like kinases observed in *Arabidopsis* do not operate in tomato plants.

**Figure 3:**
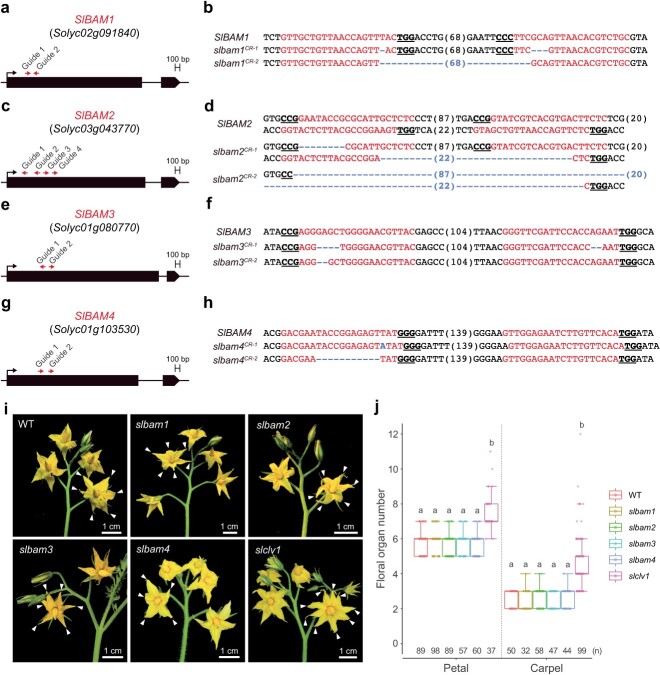
Generation of four tomato *slbam* mutants by CRISPR mutagenesis. (a) Gene structures of *SlBAM1*. (b) CRISPR-generated mutations of *SlBAM1*. (c) Gene structures of *SlBAM2*. (d) CRISPR-generated mutations of *SlBAM2*. (e) Gene structures of *SlBAM3*. (f) CRISPR-generated mutations of *SlBAM3*. (g) Gene structures of *SlBAM4*. (h) CRISPR-generated mutations of *SlBAM4*. Guide RNA and PAM sequences are highlighted. Numbers in parentheses represent gap lengths. (i) Inflorescence of WT, *slbam1*, *slbam2*, *slbam3*, *slbam4*, and *slclv1* plants. White arrowheads represent petals. (j) Quantification of floral organ (petal and carpel) numbers of WT, *slbam1*, *slbam2*, *slbam3*, *slbam4*, and *slclv1* plants. Box plots, 25th–75th percentile; center line, median; whiskers, full data range. The letters on the box plots indicate the significance groups at *P* < 0.05 (one-way ANOVA and Tukey test). Different letters between genotypes represent statistical significance. At least twice experiments were repeated independently with similar results.

**Figure 4:**
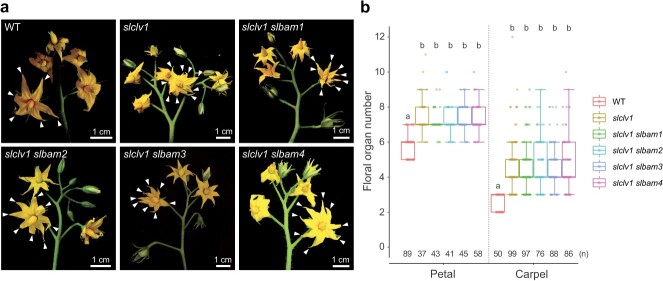
Phenotypic comparison of *slclv1* single and four *slclv1 slbam* double mutants. (a) Inflorescence of WT, *slclv1*, *slclv1 slbam1*, *slclv1 slbam2*, *slclv1 slbam3*, and *slclv1 slbam4* plants. White arrowheads indicate petals. (b) Quantification of floral organ (petal and carpel) numbers of WT, *slclv1*, *slclv1 slbam1*, *slclv1 slbam2*, *slclv1 slbam3*, and *slclv1 slbam4* plants. Box plots, 25th–75th percentile; center line, median; whiskers, full data range. The letters on the box plots signify the significance groups at *P* < 0.05 (one-way ANOVA and Tukey test). Different letters between genotypes represent statistical significance. At least twice experiments were repeated independently with similar results.

In order to examine potential genetic relationship between *SlCLV1* and its paralogs *SlBAMs*, we generated *slbam1, slbam2, slbam3,* and *slbam4* single homozygous mutant plants (Fig. 3A–H). All the *slbam* single mutants produced a normal number of floral organs in contrast to *slclv1* single mutants (Fig. 3I and J). We next generated double mutants deficient in both *SlCLV1* and a single *SlBAM* member (Fig. 4). Phenotypic analysis revealed that further loss of any single *SlBAM* gene in the *slclv1* mutant background did not increase the number of floral organs (Fig. 4A and B), requiring higher-order receptor mutants for genetic analyses.

### The *slclv1 slbam1 slbam2* triple mutants exhibit extreme fasciation

To further examine genetic redundancy between *SlCLV1* and *SlBAMs*, we generated higher-order receptor mutants. The *slbam1 slbam2* double mutants produced a similar number of floral organs compared to wild-type plants (Supplementary Fig. S2A and B). However, the plant and leaf size of *slbam1 slbam2* double mutants were dramatically reduced compared to that of wild-type plants (Supplementary Fig. S2C), mirroring observations in *Arabidopsis bam1 bam2* double mutants [21]. Thus, physiological functions of SlCLV1, SlBAM1, and SlBAM2 are not entirely identical throughout the whole plant body as observed in *Arabidopsis*.

**Figure 5:**
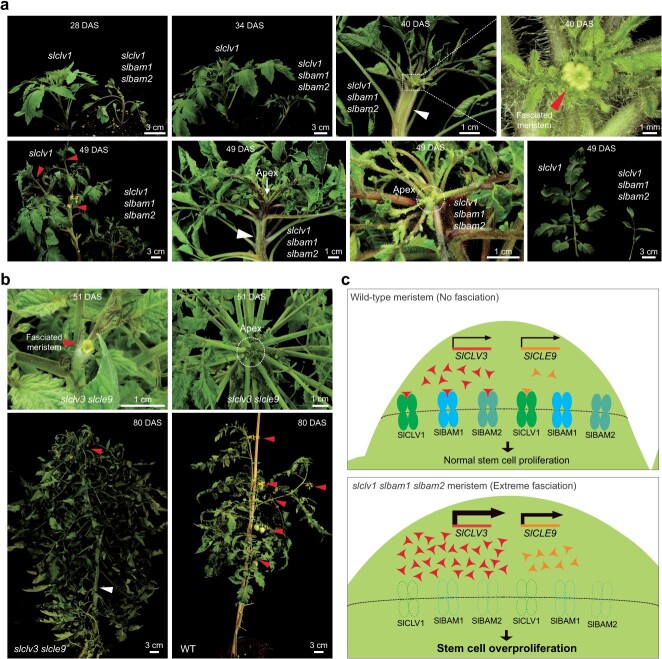
Phenotypic characterization of *slclv1 slbam1 slbam2* triple mutant plants and *slclv3 slcle9* double mutant plants. (a) Time-course images for *slclv1* single and *slclv1 slbam1 slbam2* triple mutant plants. (b) Images for WT and *slclv3 slcle9* double mutants. Arrowheads indicate fasciated stems, floral/inflorescence meristems, and inflorescences. (c) A proposed model of ligand-receptor relationship for shoot apical meristem proliferation in WT and *slclv1 slbam1 slbam2* triple mutant plants. Arrowheads represent SlCLV3 and SlCLE9 dodecapeptides. More arrowheads in the *slclv1 slbam1 slbam2* meristem indicate upregulation of *SlCLV3* and *SlCLE9* compared to wild-type meristem. At least twice, experiments were repeated independently with similar results.

Notably, the shoot meristem and primary shoot of *slclv1 slbam1 slbam2* triple mutants were more severely fasciated compared to *slclv1* single mutants and *slbam1 slbam2* double mutants (Fig. 5A, Supplementary Fig. S2D). These observations indicate that SlBAM1 and SlBAM2 partially complement SlCLV1 during stem cell maintenance when functional SlCLV1 is absent. Considering the phenotypes of double mutants lacking both *SlCLV1* and single *SlBAM* gene (Fig. 4A and B), the loss of any single *SlBAM* gene is fully compensated by remaining *SlBAM* genes even in the absence of *SlCLV1*. The phenotypic severity of *slclv1 slbam1 slbam2* triple mutants was comparable to what was observed in *slclv3 slcle9* double mutants deficient in peptide ligands (Fig. 5B). These observations support that dodecapeptides are perceived by both SlCLV1 and SlBAM receptors to control meristem proliferation (Fig. 5C).

We showed that the expression of *SlCLV1* and *SlBAM* genes did not increase significantly in *slclv1* mutants (Fig. 2E). In addition, we examined the expression of *SlCLV1* and four *SlBAM* genes in *slbam1*, *slbam2*, *slbam3*, *slbam4*, *slclv1 slbam1*, *slclv1 slbam2*, *slclv1 slbam3*, *slclv1 slbam4,* and *slclv1 slbam1 slbam2* mutants. Gene expression analysis revealed that transcript levels of *SlCLV1* and *SlBAM* genes did not exhibit by more than two-fold in these mutants (Supplementary Fig. S2E). Efficient buffering of SlCLV1 function by SlBAM1 and SlBAM2, without their expression changes, indicates strong passive compensation mechanisms between SlCLV1 and SlBAM receptors during stem cell homeostasis. In addition to shoot and meristem fasciation, *slclv1 slbam1 slbam2* triple mutants were considerably smaller than *slclv1* mutants (Fig. 5A). In contrast, *slclv3 slcle9* double mutants did not exhibit such phenotypes (Fig. 5B). Hence, it is probable that SlBAM receptors not only recognize SlCLV3 but also other peptides, contributing to the modulation of various developmental processes in tomato plants.

Next, we generated *slclv1 slbam1 slbam4* triple mutants to examine genetic redundancy between SlBAM receptors. Interestingly, *slclv1 slbam1 slbam4* triple mutants produced a similar number of floral organs compared to *slclv1* single, *slclv1 slbam1* double*,* and *slclv1 slbam4* double mutants, indicating that *slbam2* alleles result in more robust enhancement of fasciation phenotypes than *slbam4* alleles (Supplementary Fig. S3A and B). This is further supported by the extreme fasciation phenotypes of *slclv1 slbam1 slbam2 slbam4* quadruple mutants, indistinguishable to *slclv1 slbam1 slbam2* triple mutants and *slclv3 slcle9* double mutants (Supplementary Fig. S3C). Taken together, our observations suggest that unequal genetic redundancy among SlCLV1 and SlBAM receptors during perception of SlCLV3 and SlCLE9 contributes to stem cell homeostasis.

In our efforts to create mutants for the loss of four SlBAM receptors and SlCLV3 peptide, we successfully developed *slbam1 slbam4* double mutants and *slbam1 slbam4 slclv3* triple mutants for further genetic analysis. We found that *slbam1 slbam4* double mutants displayed normal carpel number like wild-type plants, whereas *slclv3* single mutants presented extra carpel number (Supplementary Fig. S4A and B) [27]. Intriguingly, carpel number of *slbam1 slbam4 slclv3* triple mutants slightly increased compared to that of *slclv3* single mutants, yet these were notably less severe than the extreme fasciation observed in *slclv1 slclv3* double mutants (Supplementary Fig. S4C) [27]. Given the known active compensation mechanisms between *SlCLV3* and *SlCLE9* [27], the less severe phenotype of *slbam1 slbam4 slclv3* triple mutants, as compared to *slclv1 slclv3* double mutants, suggests that SlCLE9 is primarily detected by SlCLV1 with partial detection by SlBAM1 and SlBAM4. It is also possible that the remaining functional SlBAM2 and SlBAM3 in *slbam1 slbam4 slclv3* mutants could also detect SlCLE9, potentially moderating the phenotype severity. Collectively, our genetic evidence indicates that SlBAMs and SlCLV1 differentially contribute to the perception of dodecapeptides during stem cell homeostasis.

### Evolutionary conservation of receptor compensation in groundcherry

We previously showed that evolutionary variations in the coding and promoter regions of peptide ligands in tomato and groundcherry lead to different capacities for compensation during meristem proliferation [28]. Consequently, we sought to determine whether similar variations might influence the compensation mechanisms of peptide receptors in two Solanaceae plants. Critical amino acid residues of CLV1 and BAM receptor proteins, associated with physical interaction with CLV3 and CLE dodecapeptides, were highly conserved in Solanaceae (Supplementary Fig. S5) [28, 34–36]. Additionally, multiple protein motifs including the leucine-rich repeat motif are conserved in receptor proteins of tomato and groundcherry (Supplementary Fig. S6).

**Figure 6:**
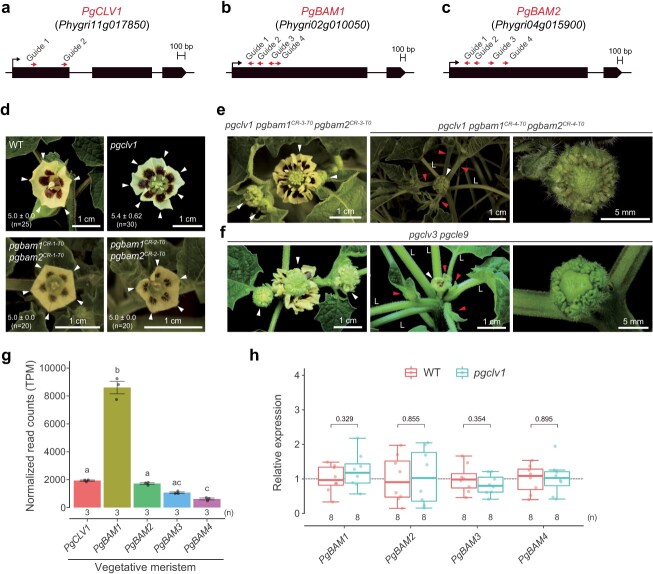
Generation and phenotypic analysis of groundcherry *pgclv1* single, *pgbam1 pgbam2* double, and *pgclv1 pgbam1 pgbam2* triple mutant plants. (a) Gene structures of *PgCLV1*. (b) Gene structures of *PgBAM1*. (c) Gene structures of *PgBAM2*. (d) Inflorescence of WT, *pgclv1*, and *pgbam1 pgbam2* T_0_ plants. Arrowheads indicate petals. The numbers in the lower left corner represent average petal numbers. (e) Inflorescence, shoot, and primary flower of CRISPR-generated *pgclv1 pgbam1 pgbam2* T_0_ plants. (f) Inflorescence, shoot, and primary flower of *pgclv3 pgcle9* double mutant plants. Arrowheads indicate flowers and shoot branches. L, leaf petioles. (g) Normalized expression for *PgCLV1*, *PgBAM1*, *PgBAM2*, *PgBAM3*, and *PgBAM4* in vegetative meristem. TPM, transcript per million. The letters on the box plots signify the significance groups at *P* < 0.05 (one-way ANOVA and Tukey test). (h) Relative expression of *PgBAM1*, *PgBAM2*, *PgBAM3*, and *PgBAM4* in shoot apices of WT and *pgclvl1* plants, normalized to groundcherry *Glyceraldehyde 3-phosphate dehydrogenase* (*PgGAPDH*)*.* Box plots, 25th–75th percentile; center line, median; whiskers, full data range. *P* values (two-tailed, two-sample *t*-test) are indicated on the box plots. Dashed line, value ‘1’ on the y-axis. Each replicate consists of eight shoot apices. Four biological replicates and two technical replicates included. At least twice experiments were repeated independently with similar results.

We thus hypothesized that passive compensation would be conserved in groundcherry that possesses *PgCLV1*, *PgBAM1*, and *PgBAM2* genes, though other *PgBAMs* could compensate passively or actively. To determine the, we employed CRISPR-Cas9 technology utilizing multiple guide RNAs to obtain *pgclv1* single, *pgbam1 pgbam2* double, and *pgclv1 pgbam1 pgbam2* triple mutants (Fig. 6A–C, Supplementary Fig. S7A–C) [37]. We first confirmed that *pgclv1* single homozygous mutants exhibited substantially milder fasciation phenotypes, consistent with the phenotype of tomato *slclv1* mutants (Figs 2C and 6D). Next, we isolated two first-generation transgenic (T_0_) plants carrying chimeric alleles of both *PgBAM1* and *PgBAM2* using a multiplex CRISPR-Cas9 construct (Fig. 6B and C). The *pgbam1 pgbam2* T_0_ plants (*pgbam1^CR-1-T0^ pgbam2 ^CR-1-T0^* and *pgbam1^CR-2-T0^ pgbam2 ^CR-2-T0^*) produced normal floral organ numbers and reduced plant and leaf size like tomato *slbam1 slbam2* double mutants (Fig. 6D, Supplementary Fig. S7D). We concurrently edited the *PgBAM1* and *PgBAM2* genes in *pgclv1* homozygous mutants and generated three *pgclv1 pgbam1 pgbam2* T_0_ plants (*pgclv1 pgbam1^CR-3-T0^ pgbam2 ^CR-3-T0^*, *pgclv1 pgbam1^CR-4-T0^ pgbam2 ^CR-4-T0^*, and *pgclv1 pgbam1^CR-5-T0^ pgbam2 ^CR-5-T0^*) that exhibited extreme fasciation phenotypes comparable to *pgclv3 pgcle9* double mutants (Fig. 6E and F, Supplementary Fig. S7E and F). These phenotypes manifested as an excessive number of floral organs and the development of additional side shoots (Fig. 6E and F).

The expression patterns of groundcherry *PgCLV1* and *PgBAMs* in shoot meristems closely resembled those of tomato *SlCLV1* and *SlBAMs*, supporting the conservation of receptor compensation between the two species (Figs 1C and 6G). Furthermore, none of the *PgBAM* genes showed an increase of more than 2-fold in the shoot apices of *pgclv1* mutants, similar to observations in tomato s*lclv1* mutants (Figs 2E and 6H). Overall, the evolutionary conservation of both critical coding sequences and passive compensation mechanisms between peptide receptors underscores robust buffering capacity during stem cell homeostasis in tomato and groundcherry.

## Discussion

### Evolutionary diversification of genetic redundancy between receptor-like kinases

Unequal genetic redundancies are frequently observed in plants. For example, *Arabidopsis ice1* single mutants are more susceptible to freezing temperature than wild-type plants, whereas freezing tolerance of *ice2* single mutants are similar to wild-type plants [38]. The *ice1 ice2* double mutants are much more vulnerable to cold stress than *ice1* single mutants, indicating that ICE2 can partially complement ICE1 which becomes evident in the absence ICE1 [38]. Thus, unequal genetic redundancies increase complexity of genetic regulation in plants depending on environmental stimuli or developmental cues [39].

In this study, we found that *slclv1* mutants showed mild fasciation and *slclv1 bam1 bam2* triple mutants exhibited extreme fasciation, whereas *slbam* mutants resembled wild-type plants, suggesting unequal genetic redundancy between SlCLV1 and SlBAM receptor-like kinases (Figs 2 and 3). This observation aligns with findings in *Arabidopsis*, where individual *bam* mutants do not display the fasciation phenotype compared to wild-type plants [21], while *clv1* null mutants show fasciated floral organs [30]. Consistent with this, previous studies revealed that *CLV1* can completely substitute for *BAM1* and *BAM2* in developing organs, but introducing *BAM1* and *BAM2* expression does not entirely substitute for *CLV1* function within the meristem in *Arabidopsis* [21]. In addition, tomato *slbam1 slbam2,* groundcherry *pgbam1 pgbam2*, and *Arabidopsis bam1 bam2* double mutants were smaller than their respective wild-type plants (Supplementary Fig. S2C and S6D) [21] Our findings indicate that in *Arabidopsis* and two Solanaceae species, the inherent functions of endogenous CLV1 and BAM receptors may differ, although they interact to regulate stem cell proliferation. Collectively, our data suggest that unequal genetic redundancy between CLV1 and BAM receptors is broadly conserved across diverse plant species.

The genetic relationship between *CLV1* and *BAMs* is not entirely identical in tomato and *Arabidopsis*. Importantly, a *clv1* mutation induces expression of *BAM* genes in *Arabidopsis* [30], while transcription of *SlCLV1* and *SlBAM* genes was not significantly increased in tomato receptor mutants (Fig. 2E, Supplementary Table S2 and Fig. S2E). This indicates that active compensation mechanisms observed in *Arabidopsis* are not conserved in tomato. The *slclv1* mutants may still have sufficient SlBAM proteins to regulate stem cell maintenance through peptide perception. Additionally, expression domains of *SlBAM* genes might shift within different cell layers of the shoot apical meristem in these mutants. As the receptor compensation could occur post-translationally, investigating how SlBAMs can partially offset the loss of SlCLV1 in stem cell regulation could provide interesting insights.

The loss of either *BAM1* or *BAM2* significantly enhances meristem defects in *Arabidopsis clv1* mutant background, with *bam1* null alleles resulting in a more substantial enhancement than *bam2* null alleles [29]. Conversely, a single mutation in *SlBAM* genes did not increase the floral organ number of *slclv1* mutants, but mutants deficient in *SlCLV1* and two members of *SlBAM* genes exhibited extreme fasciation (Fig. 5A). Therefore, in *Arabidopsis*, *BAM1* plays a more prominent role than *BAM2* in the absence of *CLV1*. These findings collectively suggest that compensation mechanisms actively buffer the severity of *clv1* mutants in *Arabidopsis*, involving unequal redundancy between BAM1 and BAM2 receptors [29]. In contrast, in tomato, *SlBAM1* and *SlBAM2* can mutually substitute for each other even in the absence of SlCLV1, suggesting that the loss of *SlCLV1* is compensated passively. Both *SlBAM1* and *SlBAM2* contribute equally to these compensation mechanisms. Thus, modes of genetic redundancy and compensation within receptor-like kinases are evolutionarily diverse, although the core receptor signaling module remains conserved.

### Conservation of receptor compensation during meristem proliferation in tomato and groundcherry

Our findings demonstrate that loss of *CLV1* homologs is passively compensated, not increasing the transcription of *BAM* homologs in two Solanaceae species, tomato and groundcherry (Figs 2E and 6H). It is noteworthy that buffering systems of meristem proliferation were significantly weakened only when both *BAM1* and *BAM2* homologous genes were absent in the tomato *slclv1* and groundcherry *pgclv1* mutants (Figs 5A and 6E). These findings suggest that a basal dosage of either the homologs of BAM1 or BAM2 receptor is adequate to completely substitute for each other and partially substitute for CLV1 homologs in tomato and groundcherry. Thus, it might be that the rate-limiting step of CLV signaling depends on the dosage of dodecapeptide ligands bound to their receptors rather than the dosage of SlBAM and PgBAM receptors in the absence of *SlCLV1* and *PgCLV1*, respectively. This is reinforced by prior findings that the absence of *SlCLV3* and *PgCLV3* peptides results in active compensation for stem cell homeostasis in tomato and groundcherry [27]. However, the loss of *CLV1* triggers active compensation by derepression and alteration of expression domains of *BAM* genes in *Arabidopsis* [30]. Interestingly, either *bam1* or *bam2* mutation significantly enhances meristem defects in *Arabidopsis clv1* mutants [29]. Therefore, it is likely that CLV signaling in *Arabidopsis* largely depends on the basal dosage of each BAM receptor in the absence of *CLV1*, unlike tomato and groundcherry. This model might explain why passive compensation mechanisms within receptor-like kinases are sufficient during stem cell maintenance in Solanaceae plants, but active compensation mechanisms are employed in *Arabidopsis*.

### Evolutionary adaptation of peptide–receptor relationship in plants

We previously demonstrated that variations in the coding region of peptide ligands determine the potency of compensation during evolution [28]. In contrast, critical amino acid residues of CLV1 and BAM receptors associated with physical interaction with dodecapeptides were highly conserved in Solanaceae (Supplementary Fig. S5). Our data suggest that SlBAM and PgBAM receptors recognize other peptide ligands as well as SlCLV3 and PgCLV3, considering the dwarf phenotypes of *slbam1 slbam2* and *pgbam1 pgbam2* double mutants (Fig. 5A, Supplementary Fig. S2C and S7D). Thus, variations in the coding region of BAM receptors might cause dysfunction in multiple peptide signaling pathways in plants, resulting in pleiotropic effects that impose significant selective pressure [40, 41]. This might explain why variations in peptide ligands are favored over receptor-like kinases during evolution.

Although most homologs of *SlCLV1* and *SlBAM* genes are widely conserved in Solanaceae, pepper and petunia lost the *SlBAM2* homolog, indicating distinguishable receptor compensation in the shoot meristem of both species (Fig. 1B). Additionally, deletion or substitution of critical amino acids for CLE dodecapeptide perception are present in the homologs of *SlBAM4* in tobacco and pepper (Supplementary Fig. S5). Our genetic data demonstrate that *SlBAM4* is marginally associated with stem cell homeostasis (Supplementary Fig. S3). This notion is also supported by our transcriptome data, which shows that *SlBAM4* was expressed at a low level in shoot meristems (Fig. 1C). It is currently unclear whether *SlBAM4* and its orthologs might have unidentified physiological functions in plants. It might be worthwhile to investigate the potential involvement of variations in *SlBAM4* and its orthologs in plant development, morphogenesis, and various stress responses.

Our data demonstrate that BAM receptors passively compensate the CLV1 receptor without transcriptional induction of these genes in two Solanaceae plants, tomato and groundcherry. To further understand how CLV1 and BAM receptors compensate for each other, creating single and multiple receptor knockout plants will be essential. Therefore, it would be worthwhile to generate receptor mutants in other Solanaceae plants and examine the conservation or variation of compensation mechanisms within Solanaceae. Our results lead to many hypotheses on how the evolutionary conservation happens for other LRR-RLKs and receptor proteins in diverse developmental and physiological contexts [12–14]. Furthermore, this work provides not only evolutionary and biological insights into genetic robustness and compensation involving plant stem cell homeostasis but also a milestone of a species-specific approach to crop improvement [42].

## Materials and methods

### Plant materials and growth conditions

Tomato cultivar ‘M82’ and groundcherry seeds originated from our collection. Both tomato and groundcherry seedlings and mature plants were cultivated in greenhouse or field conditions as previously outlined [28]. Briefly, all seeds were sown into soil and placed in a greenhouse at Kyung Hee University in Yongin, Republic of Korea, and in a greenhouse at Cold Spring Harbor Laboratory, New York, USA. Seedlings were transplanted to individual pots in the greenhouses or an agricultural field at Cold Spring Harbor Laboratory 4–5 weeks after sowing. After transplanting, both tomato and groundcherry plants were grown in the greenhouse (16 hours of light at 26–28°C, 8 hours of dark at 18–20°C, with 40–60% relative humidity), utilizing supplemental lighting from high-pressure sodium lamps and in the field. Irrigation was managed either through drip systems or overhead watering, paired with a conventional fertilization schedule. Any plants showing signs of disease or damage were identified and omitted from further data collection.

### Gene editing and plant transformation

CRISPR-Cas9-mediated mutagenesis and transformation processes for tomato and groundcherry followed established protocols [37, 43–45]. Briefly, binary vectors were assembled using Golden Gate cloning [43, 46] and then introduced into tomato and groundcherry seedlings via *Agrobacterium tumefaciens*-mediated transformation [44, 45]. Genomic DNA was extracted from at least three separate leaf samples from each T_0_ plant for analysis. The presence of transgenes and mutations induced by CRISPR were confirmed using methods previously detailed [31, 37]. Details of all primers and guide RNA sequences are contained in Supplementary Table S3.

### Plant phenotyping and imaging

All phenotypic quantification data in this study were conducted as previously described [27, 28]. Briefly, the phenotyping was performed with nontransgenic homozygous plants (tomato and groundcherry) from backcrossing or selfing and multiallelic T_0_ plants (groundcherry). To evaluate the absence of the transgenes and CRISPR-edited DNA sequences, all tomato and groundcherry mutant plants were sprayed with 400 mgl^−1^ kanamycin and genotyped by specific primers (Supplementary Table S3 and S4). All double, triple, and quadruple mutants of tomato were developed by artificial cross-pollination. We manually counted the floral organs from multiple inflorescences. At least five independent plants were used for the quantification. All the exact sample numbers are shown in figures and Supplementary Table S5. The enlarged meristem and fasciated flower images of *slclv1 slbam1 slbam2* and *pgclv1 pgbam1^CR-4-T0^ pgbam2^CR-4-T0^* mutant plants were taken using a Nikon SMZ25.

### RNA extraction, complementary DNA synthesis, quantitative real-time PCR, and transcriptome profiling

RNA extraction and quantitative real-time PCR (qPCR) for tomato and groundcherry plants followed established protocols with slight modification [28]. In brief, RNA from the shoot apices of tomato and groundcherry was extracted using the RNeasy Plant Mini Kit (Qiagen) and the PURE™ Plant RNA Extraction Kit (Infusion Tech), adhering to the provided guidelines. For cDNA synthesis, 1 μg of total RNA underwent reverse transcription using the iScript cDNA Synthesis Kit (Bio-Rad). qPCR analyses were performed with gene-specific primers (Supplementary Table S3) using the iQ SYBR Green Supermix (Bio-Rad) on a CFX96 Real-Time PCR Detection System (Bio-Rad). For each genotype, at least three shoot apices constituted a single replicate. Transcriptome data for tomato and groundcherry meristems were sourced from our previous RNA sequencing (RNA-seq) studies and available public datasets [27, 28, 47–49].

### Gene annotation, accession numbers, and phylogenetic analysis

Sequence data for tomato, potato, eggplant, pepper, tobacco, and petunia are derived from the Sol Genomics Network (https://solgenomics.net). Sequence data for *Arabidopsis* and groundcherry are derived from TAIR (https://www.arabidopsis.org/) and groundcherry genome assembly database (https://github.com/pan-sol/pan-sol-data/tree/main/Physalis), respectively [50]. *SlCLV1*, *Solyc04g081590*. *SlBAM1*, *Solyc02g091840*. *SlBAM2*, *Solyc03g043770*. *SlBAM3*, *Solyc01g080770*. *SlBAM4*, *Solyc01g103530*. *SlCIK1*, *Solyc04g039730*. *SlCIK2*, *Solyc05g005140*. *SlCIK3*, *Solyc07g006110*. *SlCIK4*, *Solyc02g072310*. *SlCIK5*, *Solyc02g089550*. *SlCIK6*, *Solyc05g010400*. *SlCIK7*, *Solyc04g005910*. *SlCLV2*, *Solyc04g056640*. *SlCRN*, *Solyc05g023760*. *SlCLV3*, *Solyc11g071380*. *SlCLE9*, *Solyc06g074060*. *SlUBQ3*, *Solyc01g056940*. *PgCLV1*, *Phygri11g017850*. *PgBAM1*, *Phygri02g010050*. *PgBAM2*, *Phygri04g015900*. *PgBAM3*, *Phygri08g029660*. *PgBAM4*, *Phygri08g009250*. *PgGAPDH*, *Phygri10g009580*. Accession numbers from other species were provided in Supplementary Table S1. For the phylogenetic analysis, MEGA-X (https://www.megasoftware.net/) was used to construct a comparative phylogenetic tree employing the Maximum Likelihood Estimation method. Bootstrap values from 1000 replicates are presented on each node.

### Statistical analyses

Statistical analyses were performed, as previously described [28]. R (RStudio version 2022.12.0+353), Microsoft Excel, and an ANOVA Calculator (https://www.statskingdom.com/180Anova1way.html#R) were utilized for our statistical computations. The statistical analysis included one-way analysis of variance (ANOVA) paired with Tukey's test and a two-tailed, two-sample *t*-test (Supplementary Table S6).

## Acknowledgements

We regret the absence of additional citations, adhering to the author guidelines that prescribe a more limited number of references. We thank all members of the Kwon lab at Kyung Hee University for comments, discussions, and assistance with plant care; all members of the Lippman lab from Cold Spring Harbor Laboratory for comments and discussions; T. Mulligan, K. Schlecht, A. Krainer, and S. Qiao from Cold Spring Harbor Laboratory for assistance with plant care; J. Van Eck, A. Horowitz Doyle, K. Swartwood, M. Tjahjadi, L. Randall, and P. Keen from Boyce Thompson Institute for assistance with the tomato and groundcherry transformations. This research was funded by the National Research Foundation (NRF) of the Ministry of Science and ICT (MSIT), Republic of Korea (No. 2022R1C1C1002941 to C.-T.K., No. 2020R1A2C1101915 to S.J.P., and No. RS-2023-00217064 to W.-J.H.), the Howard Hughes Medical Institute, and the National Science Foundation Plant Genome Research Program (No. IOS-1732253) to Z.B.L.

## Author contributions

M.-G.S. and Y.L. performed the experiments and edited the manuscript. A.H. and G.R. performed the groundcherry experiments. H.K.B. performed the tomato experiments. W.-J.H. performed the phylogenetic analysis. S.J.P. generated transgenic tomato plants and performed the tomato experiments. Z.B.L. conceived the research and supervised the experiments. Y.-J.P. performed the experiments, prepared the figures, and wrote the manuscript. C.-T.K. conceived and led the research, supervised and performed the experiments, prepared the figures, and wrote the manuscript. All authors read and approved the manuscript.

## Supplementary Data

Supplementary data is available at Horticulture Research online.

## Conflict of interest statement

The authors declare that they have no conflict of interest.

## Data availability

Raw data and information for CRISPR-generated alleles, all quantifications, and exact *P* values (one-way ANOVA and Tukey test) are in Supplementary Table S6. The raw Sanger sequence traces for edited sequences are in Supplementary Data Files. The tomato and groundcherry BioProject accession numbers are PRJNA491365, PRJNA704671, and PRJNA862958.

## Supplementary Material

Web_Material_uhae126
