# Supplementary material for: Evolutionary conservation of receptor compensation for stem cell homeostasis in Solanaceae plants
Source: Hortic Res. 2024 May 3;11(6):uhae126. doi: 10.1093/hr/uhae126 (PMC11197305; doi:10.1093/hr/uhae126)
Supplement: Web_Material_uhae126 [file web_material_uhae126.zip › HR-2024-088-Supplementary_Figures_1-7-revision_final.pdf]

Evolutionary conservation of receptor compensation for stem cell homeostasis in Solanaceae plants

Myeong-Gyun Seo[1,†], Yoonseo Lim[1,†], Anat Hendelman[2,3], Gina Robitaille[2,3], Hong Kwan Beak[4], Woo-Jong Hong[5], Soon Ju Park[6], Zachary B. Lippman[2,3], Young-Joon Park[5,*], Choon-Tak Kwon[1,5,*]

[1] Graduate School of Green-Bio Science, Kyung Hee University, Yongin 17104, Republic of Korea.

[2] Cold Spring Harbor Laboratory, Cold Spring Harbor, New York 11724, USA.

[3] Howard Hughes Medical Institute, Cold Spring Harbor Laboratory, Cold Spring Harbor, New York, NY, USA.

[4] Division of Biological Sciences and Research Institute for Basic Science, Wonkwang University, Iksan, Jeonbuk 54538, Republic of Korea.

[5] Department of Smart Farm Science, Kyung Hee University, Yongin 17104, Republic of Korea.

[6] Division of Applied Life Science, Plant Molecular Biology and Biotechnology Research Center, Gyeongsang National University, Jinju 52828, South Korea.

*Correspondence: C.-T.K. (ctkwon@khu.ac.kr) and Y.-J.P (youngjoonpark@khu.ac.kr).

[†] These authors contributed equally to this work.

Supplementary data

Figure S1. Expression levels of various tissues from *SlCLV1*, *SlBAM1*, *SlBAM2*, *SlBAM3*, and *SlBAM4*. **a** Normalized read counts of *SlCLV1* and four *SlBAM* genes from tomato cultivar "M82" tissues. **b** Normalized read counts of *SlCLV1* and four *SlBAM* genes from tomato cultivar "Heinz" tissues. All data was obtained from the tomato eFP browser (http://bar.utoronto.ca/efp_tomato/cgi-bin/efpWeb.cgi). RPKM, reads per kilobase of transcript per million mapped reads.

Figure S2. Pleiotropy of *slbam1 slbam2* double and *slclv1 slbam1 slbam2* triple mutant plants. **a** Inflorescence of WT and *slbam1 slbam2* plants. White arrowheads indicate petals. **b** Quantification of floral organ (petal and carpel) numbers of WT, *slbam1 slbam2*, and *slclv1* plants. **c** Shoot and leaf of WT and s*lbam1 slbam2* plants. White and red arrowheads mark leaves and inflorescences, respectively. **d** Shoot, inflorescence, and stem of *slbam1 slbam2* double and *slclv1 slbam1 slbam2* triple mutant plants. Dashed rectangles represent inflorescences (*slbam1 slbam2*) and floral meristem (*slclv1 slbam1 slbam2*). A white arrowhead indicates a fasciated stem. **e** Relative expression of *SlCLV1*, *SlBAM1*, *SlBAM2*, *SlBAM3*, and *SlBAM4* in shoot apices of WT, *slbam* single mutants, *slclv1 slbam* double mutants, *slclv1 slbam1 slbam2* triple mutant plants, normalized to tomato *Ubiquitin3* (*SlUBQ3*). Dashed line, value '1' on the y-axis. Each replicate consists of five shoot apices. Three or four biological replicates and two technical replicates included. Box plots, 25th–75th percentile; center line, median; whiskers, full data range in **b** and **e**. The letters on the box plots signify the significance groups at $P < 0.05$ (one-way ANOVA and Tukey test) in **b** and **e**. Different letters between genotypes represent statistical significance in **b** and **e**. At least twice experiments were repeated independently with similar results.

Figure S3. *slclv1 slbam1 slbam4* and *slclv1 slbam1 slbam2 slbam4* mutants resemble to *slclv1* and *slclv1 slbam1 slbam2* mutants, respectively. **a** Inflorescence of *slclv1 slbam1 slbam4*. White arrowheads mark petals. **b** Quantification of floral organ (petal and carpel) numbers of WT, *slclv1*, *slclv1 slbam1*, *slclv1 slbam4*, and *slclv1 slbam1 slbam4* mutants. Box plots, 25th–75th

percentile; center line, median; whiskers, full data range. The letters on the box plots indicate the significance groups at $P < 0.05$ (one-way ANOVA and Tukey test). Different letters between genotypes represent statistical significance. **c** Side and top-down views of *slclv1 slbam1 slbam2 slbam4* quadruple mutant plants showing the shoot and fasciated shoot apical meristem. White and red arrowheads indicate fasciated stem and floral meristem, respectively. At least twice experiments were repeated independently with similar results.

Figure S4. Inflorescence and shoot of *slbam1 slbam4 slclv3* and *slclv1 slclv3* mutant plants. **a** Inflorescence of *slbam1 slbam4*, *slclv3*, and *slbam1 slbam4 slclv3* mutants. **b** Quantification of carpel numbers of WT, *slclv1*, *slbam1 slbam4*, *slclv3*, and *slbam1 slbam4 slclv3* mutants. Box plots, 25th–75th percentile; center line, median; whiskers, full data range. The letters on the box plots indicate the significance groups at $P < 0.05$ (one-way ANOVA and Tukey test). Different letters between genotypes mark statistical significance. **c** Shoot and apex of *slclv1 slclv3* double mutant plants resembling *slclv3 slcle9* double mutant plants.

Figure S5. Alignment of CLV1 and BAM protein sequences in tomato, potato, eggplant, pepper, groundcherry, tobacco, petunia, and *Arabidopsis*. Red letters represent the two ultra-conserved residues highly associated with the physical interaction of the sixth residue in CLE dodecapeptides. All the sequences are from the Sol Genomics Network (solgenomics.net) and groundcherry genome assembly database (https://github.com/pan-sol/pan-sol-data/tree/main/Physalis). Detailed sequence information is shown in **Supplementary Data Table S1**.

Figure S6. Sequence analyses of *SlCLV1* and *SlBAM* homologous genes. **a** Analysis of leucine-rich repeat N-terminal (LRRNT) motif composition for SlCLV1 and SlBAM homologs. **b** Multiple Expectation Maximization for Motif Elicitation (MEME) of N-terminal region of SlCLV1 and SlBAM homologs. **c** Analysis of protein motif composition for SlCLV1 and SlBAM homologs. **d** Multiple Expectation Maximization for Motif Elicitation (MEME) of SlCLV1 and SlBAM homologs. At least twice analyses were repeated independently with the same results. All data was obtained from The MEME suite(https://meme-suite.org/meme/).

Figure S7. CRISPR-generated sequences and additional phenotype of *pgbam1 pgbam2* double T_0 and *pgclv1 pgbam1 pgbam2* triple T_0 mutant plants. **a** CRISPR-generated mutations of *PgCLV1*. **b** CRISPR-generated mutations of *PgBAM1*. **c** CRISPR-generated mutations of *PgBAM1*. The raw Sanger sequence traces are captured to show chimeric sequences. **d** Shoot of non-edited and edited *pgbam1 pgbam2* T_0 plant. **e** Side view of *pgclv1 pgbam1 pgbam2* T_0 plant. White arrowhead marks fasciation stem. **f** Flower and shoot branches of *pgclv1 pgbam1 pgbam2* T_0 plant. White and red arrowheads represent extremely a fasciated flower and multiple shoot branches, respectively. L, leaf petioles.

Table S1. Protein sequences of CLV1 and BAM homologs for phylogenetic analysis.
Table S2. Normalized counts from RNA-seq and relative expression from qPCR.
Table S3. Primers used in this study.
Table S4. CRISPR-generated mutations in this study.
Table S5. Quantification data for organ numbers in this study.
Table S6. Exact *P*-values in this study (One-way ANOVA with Tukey test).

Figure S1

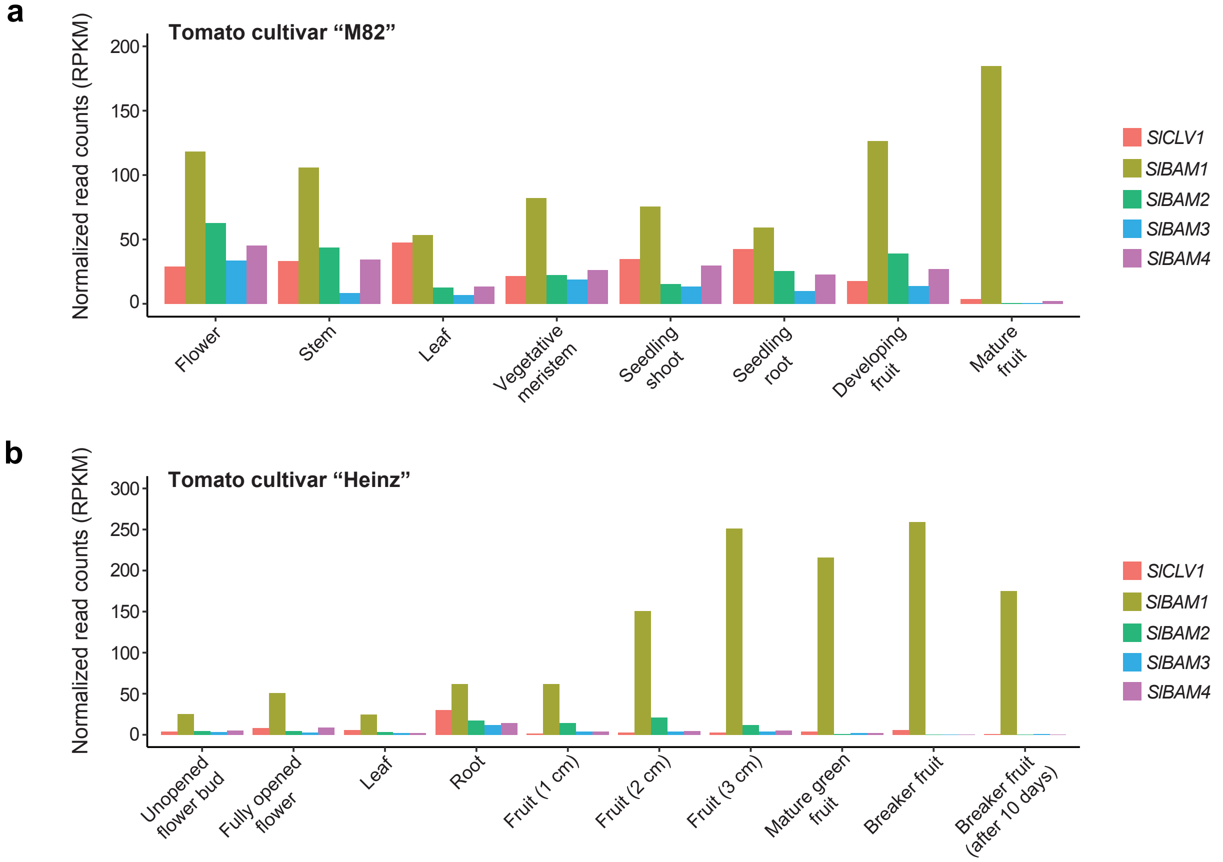
a
Tomato cultivar "M82"
Normalized read counts (RPKM)
0
50
100
150
200
Flower
Stem
Leaf
Vegetative meristem
Seedling shoot
Seedling root
Developing fruit
Mature fruit
SlCLV1
SlBAM1
SlBAM2
SlBAM3
SlBAM4
b
Tomato cultivar "Heinz"
Normalized read counts (RPKM)
0
50
100
150
200
250
300
Unopened flower bud
Fully opened flower
Leaf
Root
Fruit (1 cm)
Fruit (2 cm)
Fruit (3 cm)
Mature green fruit
Breaker fruit
Breaker fruit (after 10 days)
SlCLV1
SlBAM1
SlBAM2
SlBAM3
SlBAM4

Figure S2

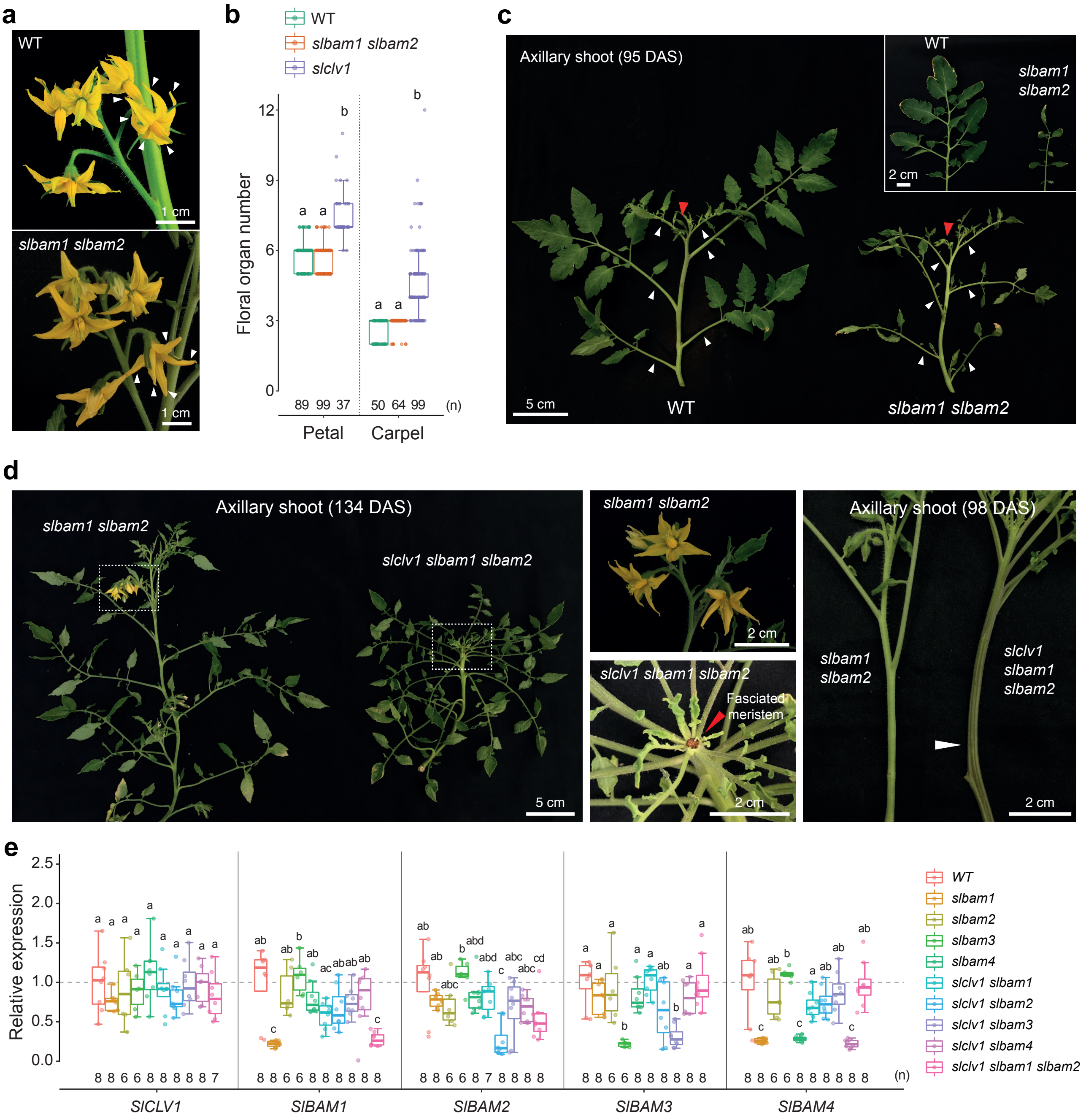
a
WT
1 cm
slbam1 slbam2
1 cm
b
WT
slbam1 slbam2
slclv1
Floral organ number
0
3
6
9
12
a a b
a a b
89 99 37
50 64 99
(n)
Petal
Carpel
c
Axillary shoot (95 DAS)
WT
slbam1 slbam2
2 cm
5 cm
WT
slbam1 slbam2
d
Axillary shoot (134 DAS)
slbam1 slbam2
slclv1 slbam1 slbam2
5 cm
slbam1 slbam2
2 cm
slclv1 slbam1 slbam2
Fasciated meristem
2 cm
Axillary shoot (98 DAS)
slbam1 slbam2
slclv1 slbam1 slbam2
2 cm
e
Relative expression
0.0
0.5
1.0
1.5
2.0
2.5
8 8 6 6 8 8 8 8 8 7
8 8 6 6 8 8 8 8 8 8
8 8 6 6 8 7 8 8 8 8
8 8 6 6 8 8 8 8 8 8
8 8 6 6 8 8 8 8 8 8
(n)
SlCLV1
SlBAM1
SlBAM2
SlBAM3
SlBAM4
WT
slbam1
slbam2
slbam3
slbam4
slclv1 slbam1
slclv1 slbam2
slclv1 slbam3
slclv1 slbam4
slclv1 slbam1 slbam2

Figure S3

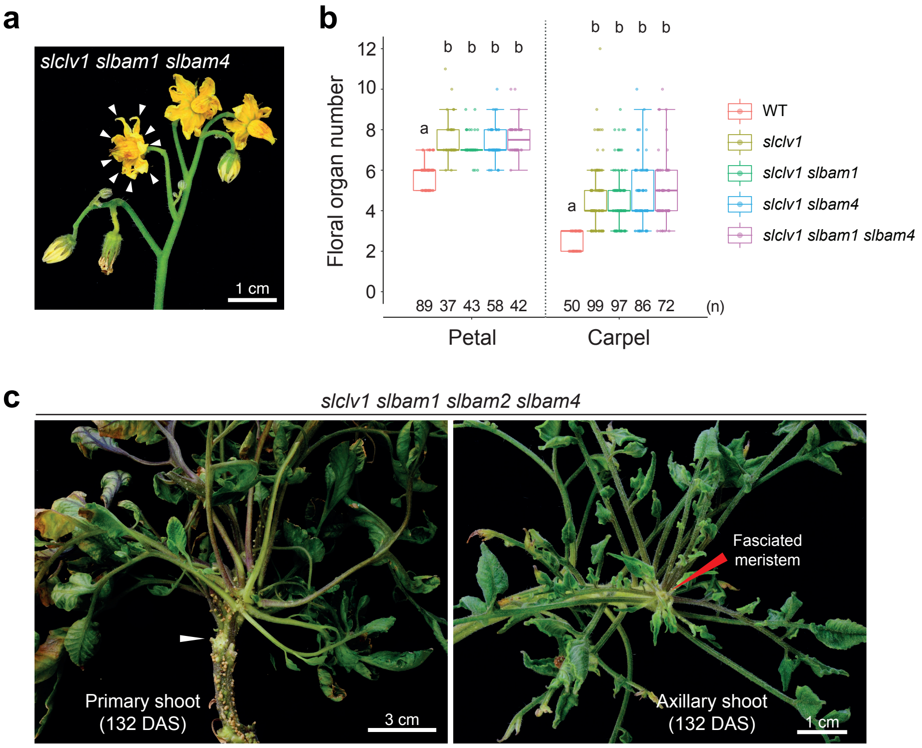
a
slclv1 slbam1 slbam4
1 cm
b
Floral organ number
12
10
8
6
4
2
0
a
b b b b
a
b b b b
89 37 43 58 42
50 99 97 86 72
(n)
Petal
Carpel
WT
slclv1
slclv1 slbam1
slclv1 slbam4
slclv1 slbam1 slbam4
c
slclv1 slbam1 slbam2 slbam4
Primary shoot
(132 DAS)
3 cm
Fasciated
meristem
Axillary shoot
(132 DAS)
1 cm

Figure S4

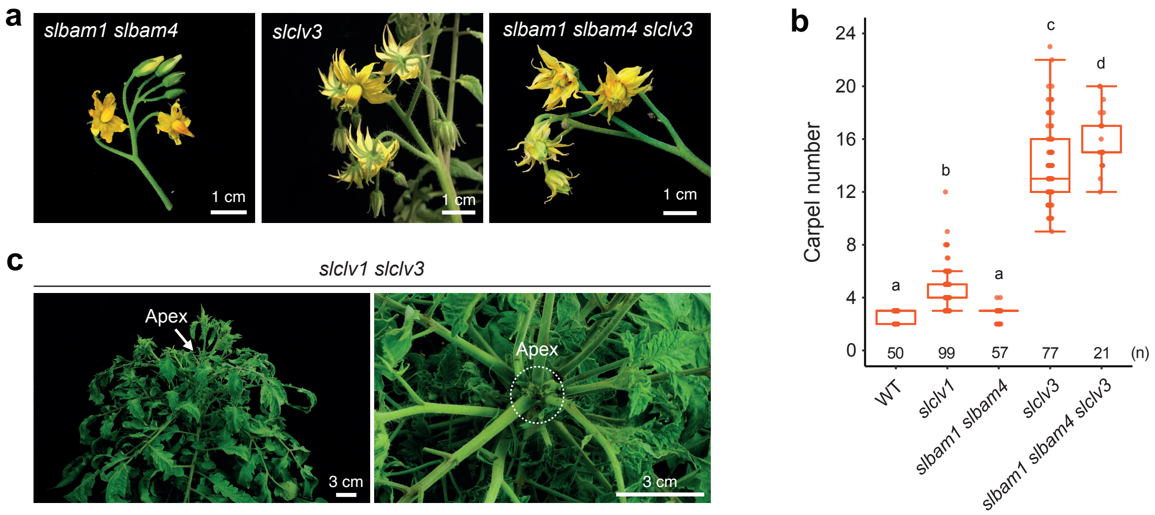
a
slbam1 slbam4
1 cm
slclv3
1 cm
slbam1 slbam4 slclv3
1 cm
b
Carpel number
24
20
16
12
8
4
0
a
b
a
c
d
50
99
57
77
21
(n)
WT
slclv1
slbam1 slbam4
slclv3
slbam1 slbam4 slclv3
c
slclv1 slclv3
Apex
3 cm
Apex
3 cm

Figure S5

```
Solyc04g081590(SlCLV1)      LLPNLEELRLGYYNSYEGGIPSEFGNISTLKLLDLGNCNLDGEVPPSLGNLKKLHSLFLQ  273
Solyc02g091840(SlBAM1)      NIATLQQLYVGYYNTFTGGIPPAIGNLSQLLRFDAANCGLSGKIPPEIGKLQNLDTLFLQ  267
Solyc03g043770(SlBAM2)      NITTLRELYIGYYNTFSGGLPAEIGYLSELIRLDAANCGLSGEIPPEIGKLQKLDTLFLQ  271
Solyc01g080770(SlBAM3)      NVTSLRWLQLGYYNQFDEGIPPELGKLVNLVHLDLSSCNLTGSIPPELGNLNMLDTLFLQ  253
Solyc01g103530(SlBAM4)      NLTNLKEIYLGYFNVFVGGIPKEFGKLENLVHMDISNCELDGPIPPELGNLKLLNTLFLH  262
Soltu.DM.04G036750          SLPNLEELRLGYYNSYEGGIPSEFGNISTLKLLDLGNCNLDGEVPPSLGNLKKLHTLFLQ  272
Soltu.DM.02G027100          NITTLQQLYVGYYNTFTGGIPPAIGNLSQLLRFDAANCGLSGEIPPEIGKLQNLDTLFLQ  267
Soltu.DM.03G005050          NITTLRELYIGYYNTFSGGLPAEIGNLSELIRLDAANCGLSGEIPPEIGKLQKLDTLFLQ  271
Soltu.DM.01G023180          NVTSLRWLQLGYYNQFDEGIPPELGKLVNLVHLDLSSCNLTGSIPAELGNLNMLDTLFLQ  253
Soltu.DM.01G042460          NLTNLKEIYLGYFNVFVGGIPKEFGKLENLVHMDISNCELDGPIPPELGNLKLLNTLFLH  266
SMEL4.1_04g023660           LLPNLEELKLGYFNSYEGGIPSEFGSISTLKLLDLGNCNLEGEIPPSLGNLKKLHTLFLQ  271
SMEL4.1_02g024600           NITTLQQLYVGYYNTFSGGIPPEIGNLSHLIRFDAANCGLSGEIPPEIGKLQNLDTLFLQ  269
SMEL4.1_03g004650           NISTLRELYIGYYNTFSGGLPPEIGNLSELIRLDAANCGISGEIPPEIGKLQKLDTLFLQ  266
SMEL4.1_01g025440           NLTNLRWLKLGYYNQFEQGIPPELGKLINLVHLDLSSCNLTGPIPAELGNLHMLDTLFLQ  252
SMEL4.1_01g000680           NLTNLKEIYLGYYNVFVGGIPKEFGKLENLVHMDISNCELDGPIPPELGNLKLLNTLFLH  280
CA04g21950                  LLPNLEILKLGYFNSYEGGIPPEFGNISTLKLLDLGSCNLDGEIPPSLANLKKLHSLFLQ  275
CA02g24570                  NISTLQQLYVGYYNNFSGGIPPEIGNLTGLQRFDAANCGLSGVIPPEIGKLQNLDTLFLQ  266
CA01g25290                  NLTNLRWLQLGYFNQFDEGIPPELGKLVNLVHLDLSSCNLMGPIPAELGNLNMLDTLFLQ  257
CA08g15290                  NLTNLKEIYLGYYNVFVGGIPKEFGKLENLVHMDISSCELDGPIPPELGNLKLLNTLYLH  274
Phygri11g017850             LLPNLEELRLGYYNSYEGGIPPEFGSISTLKYLDLGSCNLDGEIPPSLGNLKKMHTLFLQ  273
Phygri02g010050             NITTLQQLYVGYYNTFSGGIPPEIGNLSQLLRFDAANCGISGEIPPEIGKLQNLDTLFLQ  266
Phygri04g015900             NITSLRELYIGYYNTFSGGLPPEIENLSELNRLDAANCGLSGEIPPEIGKLQKLDTLFLQ  268
Phygri08g029660             NLTNLRWLQLGYFNQFDEGIPPELGKLANLVHLDLSSCNLTGPIPAELGNLNMLDTLFLQ  257
Phygri08g009250             NLTNLKEIYLGYFNVFVGGIPKEFGKLENLVHMDISSCELDGPIPPELGNLKLLHTLFLH  273
Niben101Scf09924g01002      LLPNLEELRLGYFNSYEGGIPTEFGSISTLKLLDLANCNLDGEIPPSLGNLKKLHSLFLH  276
Niben101Scf02740g12007      NITTLKELYVGYYNAFSGGLPAAIGNLSELVRLDAANCRLSGEIPAEIGKLKKLDTLFLQ  265
Niben101Scf00904g03002      NITTLKELYVGYYNAFSGGIPPEIGNLSELVRFDAANCGLSGEIPPEIGKLKKLDTLFLQ  266
Niben101Scf09708g00005      NLTNLRWLQLGYFNQFDGGIPPELGKLVNLVHLDLSSCNLDGSIPAELGNLNMLDTLFLQ  257
Niben101Scf04197g01023      NLTNLKEIFLGYFNVFVGGIPKEFGKLENLVHMDISSCELDGPIPPELGNLKLLNTLFLH  269
Niben101Scf02417g06008      NITTLHELYVGYYNTFSGGIPPEIGNLSQLVRFDAANCGLSGEIPAEIGKLQNLDTLFLQ  118
Niben101Scf04253g03010      NVTTLHELYVGYYNTFSGGIPPEIGNLSQLVRFDAANCGLSGEIPAEIGKLQNLDTLFLQ  269
Niben101Scf01711g01005      NLTSLKEIYLGYFNVFVGQIPKELGN----------------------------------  235
Niben101Scf14837g01005      NLTNLRWLQLGYFNQFDKGIPPELGKLVNLVHLDLSSCNLDGSIPAELGILKMLDTLFLQ  251
Peinf101Scf00070g09009      LLPNLEELRLGYYNSYEGGIPPEFGSISTLKLLDLGSCNLDGEIPASLGNLKKLHSLFLQ  270
Peinf101Scf00578g00008      NISSLKELYLGYYNTFSGGIPPEIGNLSNLVRLDAANCGLSGEIPQEIGKLQNLDTLFLQ  268
Peinf101Scf00504g15012      NLTNLRWLQLGYFNQFDGGIPAELGKLVNLVHLDLSSCNLKGPIPVELGNLNMLDTLFLQ  261
Peinf101Scf02714g01043      NLTNLKEIYLGYFNVFVGGIPKEFGKLENLVHMDISSCELDGPIPPELGNLKLLNTLFLH  268
Peaxi162Scf00553g00620      LLPNLEELRLGYYNSYEGGIPPEFGSITTLKLLDLGSCNLDGEIPASLGNLKKLHSLFLQ  270
Peaxi162Scf00096g01311      NISSLKELYLGYYNTFSGGIPPEIGNLSNLVRLDAANCGLSGEIPQEIGKLQNLDTLFLQ  268
Peaxi162Scf00736g00045      NLTNLRWLQLGYFNQFEGGIPAELGKLVNLVHLDLSSCNLKGPIPVELGNLNMLDTLFLQ  261
Peaxi162Scf00011g01324      NLTNLKEIYLGYFNVFVGGIPKEFGKLENLVHMDISSCELDGPIPPELGNLKLLNTLFLH  268
AT1G75820(AtCLV1)           RLKNLREMYIGYYNSYTGGVPPEFGGLTKLEILDMASCTLTGEIPTSLSNLKHLHTLFLH  274
AT5G65700(AtBAM1)           NLTTLRELYIGYYNAFEDGLPPEIGNLSELVRFDGANCGLTGEIPPEIGKLQKLDTLFLQ  271
AT3G49670(AtBAM2)           NLTTLRELYIGYYNAFENGLPPEIGNLSELVRFDAANCGLTGEIPPEIGKLQKLDTLFLQ  271
AT4G20270(AtBAM3)           NITTLVQLYLGYYNDYRGGIPADFGRLINLVHLDLANCSLKGSIPAELGNLKNLEVLFLQ  279
                            ::::*..:.:**:*.:.:::*.:.:.:..:..::..::.:.:.::.::::..:.::::::
```

Figure S6

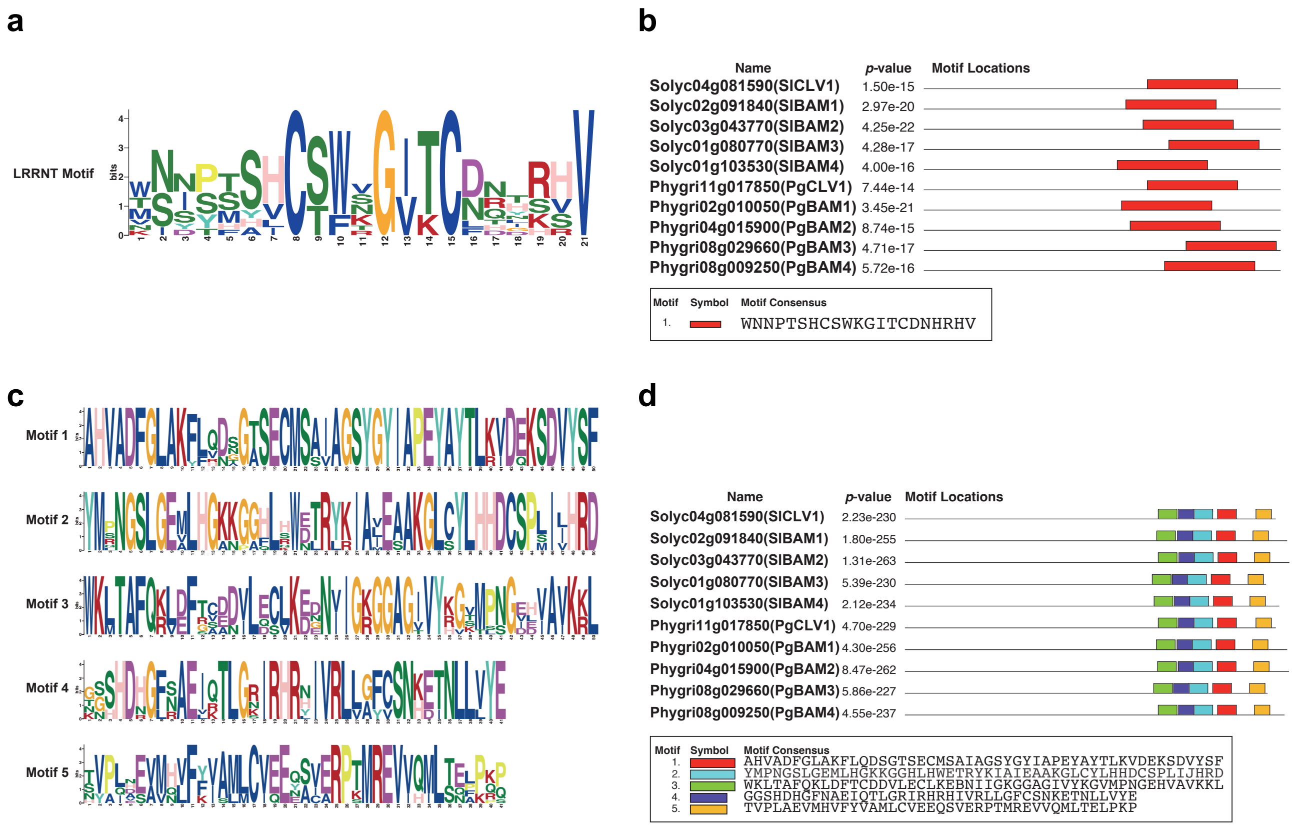
a
LRRNT Motif
bits
b
Name
p-value
Motif Locations
Solyc04g081590(SlCLV1) 1.50e-15
Solyc02g091840(SlBAM1) 2.97e-20
Solyc03g043770(SlBAM2) 4.25e-22
Solyc01g080770(SlBAM3) 4.28e-17
Solyc01g103530(SlBAM4) 4.00e-16
Phygri11g017850(PgCLV1) 7.44e-14
Phygri02g010050(PgBAM1) 3.45e-21
Phygri04g015900(PgBAM2) 8.74e-15
Phygri08g029660(PgBAM3) 4.71e-17
Phygri08g009250(PgBAM4) 5.72e-16
Motif
Symbol
Motif Consensus
1. WNNPTSHCSWKGITCDNHRHV
c
Motif 1
Motif 2
Motif 3
Motif 4
Motif 5
d
Name
p-value
Motif Locations
Solyc04g081590(SlCLV1) 2.23e-230
Solyc02g091840(SlBAM1) 1.80e-255
Solyc03g043770(SlBAM2) 1.31e-263
Solyc01g080770(SlBAM3) 5.39e-230
Solyc01g103530(SlBAM4) 2.12e-234
Phygri11g017850(PgCLV1) 4.70e-229
Phygri02g010050(PgBAM1) 4.30e-256
Phygri04g015900(PgBAM2) 8.47e-262
Phygri08g029660(PgBAM3) 5.86e-227
Phygri08g009250(PgBAM4) 4.55e-237
Motif
Symbol
Motif Consensus
1. AHVADFGLAKFLQDSGTSECMSAIAGSYGYIAPEYAYTLKVDEKSDVYSF
2. YMPNGSLGEMLHGKKGGHLHWETRYKIAIEAAKGLCYLHHDCSPLIJHRD
3. WKLTAFQKLDFTCDDVLECLKEBNIIGKGGAGIVYKGVMPNGEHVAVKKL
4. GGSHDHGFNAEIQTLGRIRHRHIVRLLGFCSNKETNLLVYE
5. TVPLAEVMHVFYVAMLCVEEQSVERPTMREVVQMLTELPKP

Figure S7

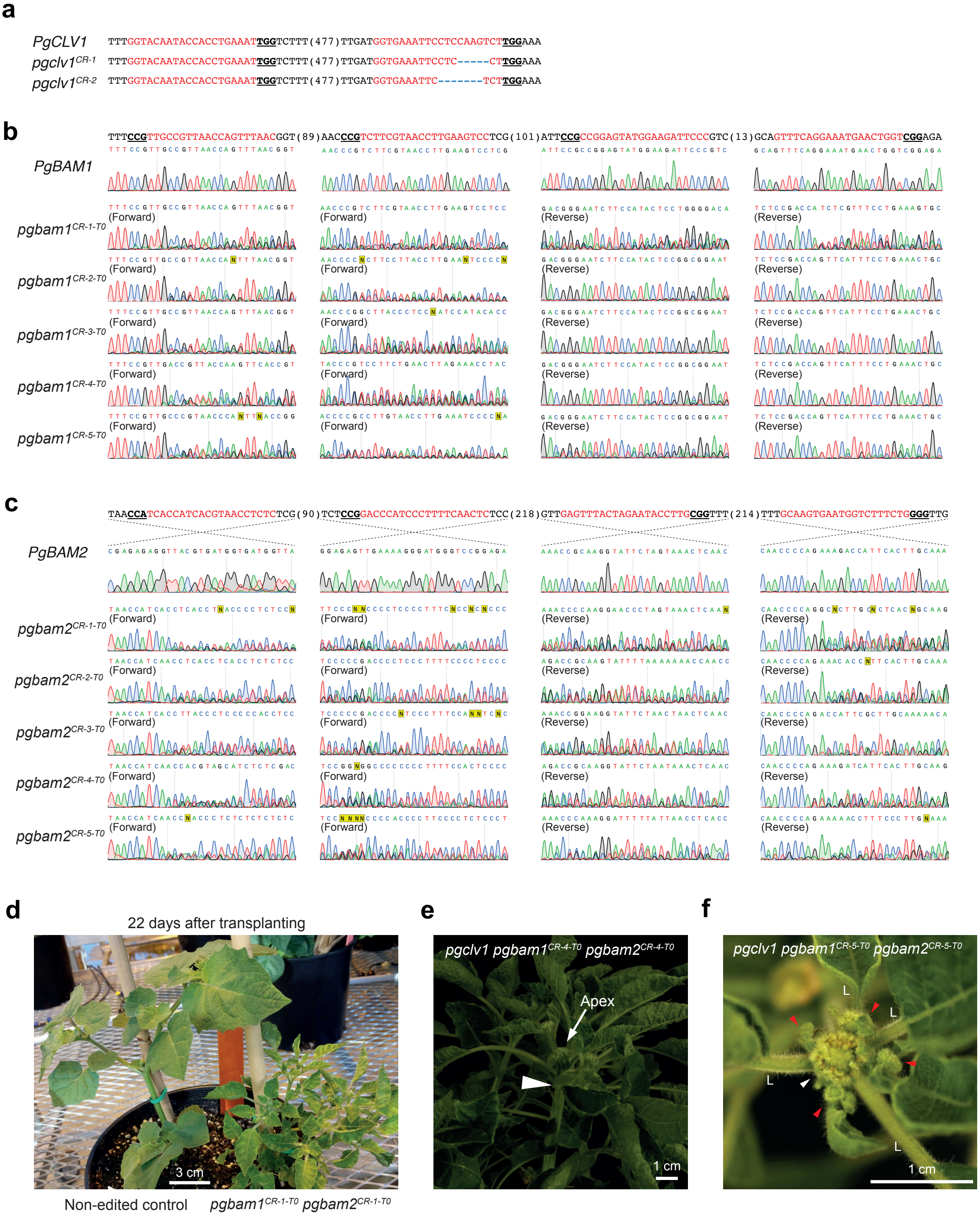
a
PgCLV1 TTTGGTACAATACCACCTGAAATTGGTCTTT(477)TTGATGGTGAAATTCCTCCAAGTCTTGGAAA
pgclv1CR-1 TTTGGTACAATACCACCTGAAATTGGTCTTT(477)TTGATGGTGAAATTCCTC-----CTTGGAAA
pgclv1CR-2 TTTGGTACAATACCACCTGAAATTGGTCTTT(477)TTGATGGTGAAATTC-------TCTTGGAAA
b
TTTCCGTTGCCGTTAACCAGTTTAACGGT(89)AACCCGTCTTCGTAACCTTGAAGTCCTCG(101)ATTCCGCCGGAGTATGGAAGATTCCCGTC(13)GCAGTTTCAGGAATGAACTGGTCGGAGA
PgBAM1
pgbam1CR-1-T0
pgbam1CR-2-T0
pgbam1CR-3-T0
pgbam1CR-4-T0
pgbam1CR-5-T0
(Forward)
(Reverse)
c
TAACCATCACCATCACGTAACCTCTCTCG(90)TCTCCGGACCCATCCCTTTTCAACTCTCC(218)GTTGAGTTTACTAGAATACCTTGCGGTTT(214)TTTGCAAGTGAATGGTCTTTCTGGGTTG
PgBAM2
pgbam2CR-1-T0
pgbam2CR-2-T0
pgbam2CR-3-T0
pgbam2CR-4-T0
pgbam2CR-5-T0
(Forward)
(Reverse)
d
22 days after transplanting
3 cm
Non-edited control
pgbam1CR-1-T0 pgbam2CR-1-T0
e
pgclv1 pgbam1CR-4-T0 pgbam2CR-4-T0
Apex
1 cm
f
pgclv1 pgbam1CR-5-T0 pgbam2CR-5-T0
L
1 cm
